# Rehydration of the sleeping chironomid, *Polypedilum vanderplanki* Hinton, 1951 larvae from cryptobiotic state up to full physiological hydration (Diptera: Chironomidae)

**DOI:** 10.1038/s41598-022-07707-1

**Published:** 2022-03-08

**Authors:** Stanisław Knutelski, Hubert Harańczyk, Piotr Nowak, Andrzej Wróbel, Bartosz Leszczyński, Takashi Okuda, Kazimierz Strzałka, Ewelina Baran

**Affiliations:** 1 Institute of Zoology and Biomedical Research, Jagiellonian University, Kraków, Poland; 2 Institute of Physics, Jagiellonian University, Kraków, Poland; 3 Faculty of Computer Science, Electronics and Telecommunications, AGH University of Science and Technology, Kraków, Poland; 4 Nemli Project LLC, Tsuchiura, Japan; 5 Faculty of Biochemistry, Biophysics and Biotechnology, Jagiellonian University in Krakow, Kraków, Poland; 6 Malopolska Centre of Biotechnology, Jagiellonian University in Krakow, Kraków, Poland; 7 Institute of Technology, Pedagogical University of Krakow, ul. Podchorążych 2, 30-084 Kraków, Poland

**Keywords:** Biological techniques, Biophysics, Physics

## Abstract

During desiccation the *Polypedilum vanderplanki* larva loses 97% of its body water, resulting in the shutdown of all metabolic and physiological processes. The larvae are able to resume active life when rehydrated. As dehydration process has already been largely understood, rehydration mechanisms are still poorly recognized. X-ray microtomograms and electron scanning microscopy images recorded during the hydration showed that the volume of the larva's head hardly changes, while the remaining parts of the body increase in volume. In the ^1^H-NMR spectrum, as recorded for active larvae, component characteristic of solid state matter is absent. The spectrum is superposition of components coming from tightly and loosely bound water fraction, as well as from lipids. The value of the c coefficient (0.66 ± 0.02) of the allometric function describing the hydration models means that the increase in the volume of rehydrated larvae over time is linear. The initial phase of hydration does not depend on the chemical composition of water, but the amount of ions affects the further process and the rate of return of larva’s to active life. Diffusion and ion channels play a major role in the permeability of water through the larva's body integument.

## Introduction

Living organisms use different types of adaptation strategies to survive unfavorable environmental conditions, such as: extremely low and high temperatures, limited access to food, high concentration of salts or heavy metals, oxidation and hypoxia, or dehydration. One of the most serious environmental stresses, both for aquatic and for terrestrial organisms is the lack of water or its limited access^1,2^.

Water is one of the essential molecules allowing life of living organisms on the Earth^1,3^ and the most mysterious liquid that has been known so far in terms of physical and chemical properties^2^. Water usually accounts for approximately 60–90% of the mass of organisms. In invertebrates the average amount of body water is around 70% (from 17 to 90%) and it is fundamental for all its vital reactions, water makes up 95–99% of the total number of molecules^3^. Its deficit in extreme environments leads to dehydration of organisms, which is one of the most serious problems for the Earth’s life and can cause serious damage or even death^3^. Small invertebrate organisms, such as insects, losing water easily due to relatively large body surface per volume^4,5^, and relatively being especially vulnerable to dehydration, must preserve a body’s water content and water concentration within tolerable limits in order to maintain physiological integrity^6,7^. Existence of freshwater rock pools, the oldest freshwater habitats worldwide^8^, and particularly periodically drying out pools in semi-arid areas in Africa, seem relatively should be very threatened in the current global conditions of nature^9,10^. In the context of global environmental change such changing temperatures, patterns of rainfall and water availability, understanding the responses of insects to water availability is important, especially, because it has a pronounced influence on insect activity, distribution patterns, and species richness^11^.

Some organisms experiencing the exposure to dry conditions have evolved various adaptive physiological strategies and the molecular mechanisms to combat desiccation and survive at extreme environmental conditions without rainfall for months thanks to the dehydration–rehydration cycle^12–19^. The most sophisticated example of peculiar biological state in which some organisms showing no sign of life due to almost complete loss of body water and abatement of their metabolic activity to an undetectable level is a phenomenon named anhydrobiosis^20,21^, which seems to be a state between life and death^3^.

Despite the presence of anhydrobiosis in species belonging to several distant evolutionary lines and its adaptive potentiality, in metazoans anhydrobiosis can be found only in a restricted number of taxa whose animal sizes do not exceed 5–7 mm, and often it is much smaller^22^. The majority of the Earth’s organisms having the ability to convert to anhydrobiosis and then return to full life functions do not exceed 1 mm of body size^22^. The exception are the aquatic larvae of the sleeping chironomid or african sleeping chironomid, *Polypedilum vanderplanki* Hinton 1951, true euryhygrobiote dipterans in the family Chironomidae, which reach 6–7 mm in length^23–25^. The species represents the largest multicellular organism and the most complex animal with anhydrobiotic ability, the only insect that can enter anhydrobiosis, an ametabolic state, and withstand near complete desiccation in order to survive adverse environmental conditions such as periodic drought^13,21–25^. This species seems currently threatened with extinction due to intensive catches of its larvae by the local inhabitants for commercial purposes. In the period of sufficient water access the larvae of *P. vanderplanki* live in tubular nests made by detritus and saliva in shallow temporary pools on rocks in semi-arid areas on the African continent. During periodic drying of these pools the larvae hide in small tubular nests in the mud at the bottom. Upon drought the larval tubes can maintain a high relative humidity inside and potentially slow down the rate of water loss from the larva^26^. During desiccation the *P. vanderplanki* larva loses 97% of its body water^19^, resulting in the shutdown of all metabolic and physiological processes and become completely desiccated but are able to resume active life of all cells without obvious damage after rehydration when water becomes available upon the next rain^12,21,27,28^. During the anhydrobiotic period, the larvae upregulate various gene expressions^14,26,29–31^ and accumulate trehalose up to ca. 20% of their dry weight^32^, and accumulate LEA-like proteins^29^, exhibiting extremely high tolerance^27^ not only for desiccation, but also for many other types of environmental stress such as: high and low temperatures, the absence of oxygen, radiation (including ultraviolet radiation), enhanced radioresistance, high hydrostatic pressure, and chemical stresses^25,27,33–35^. They also tolerate repeated dehydration^22,25^. Gusev et al.^36^ found that dehydration causes alterations in chromatin structure and a severe fragmentation of nuclear DNA in the cells of the African sleeping chironomid larvae despite successful anhydrobiosis.

In order to achieve anhydrobiotic state successfully, *P. vanderplanki* larvae need a slow dehydration process taking at least 2 days^26^. However, when the dried larvae are immersed in water, their metabolism is resumed and rehydrated larvae revive in less than 1 h and become almost indistinguishable from non dehydrated individuals^37^.

Factors responsible for desiccation tolerance in *P. vanderplanki* larvae and mechanisms underlying their induction during desiccation are relatively well studied, but a little is known about their rehydration^3,12–14,16,22,23,25–30,33,35–37^. Generally, our current knowledge represents only a little information on rehydration in insects^6,38^. Rehydration is the process of restoring water content into someone's body when they are suffering from dehydration (= a lack of water). It seems equally important as desiccation one to know the process and mechanisms of rehydration and the return of the organism to the full function of life from the state of anhydrobiosis in the context of the survival of organisms in extremely unfavorable environmental conditions associated with periodic lack of water.

We present and describe new data about the rehydration process of *Polypedilum vanderplanki* larvae. Using advanced research methods: Microtomography (micro-CT), Scanning Electron Microscope (SEM), Nuclear Magnetic Resonance (^1^H NMR), and Morphometry by stereoscopic microscope, we could observe and analyze the morphological consequences accompanying this phenomenon and the way and rate at which water enters the body in various ex situ environmental conditions from anhydrobiotic state to full hydration, manifested by full larvae life activity.

## Materials and methods

### Materials

Anhydrobiotic (= dry) larvae of the sleeping chironomid, *Polypedilum vanderplanki* Hinton, 1951 (Insecta; Diptera: Chironomidae) were collected by Dr. Okuda from rock pools in Nigeria andtransported to the laboratory at NIAS (Japan), where they were reared according to the Kikawada et al.^26^ procedure. Anhydrobiotic larvae of a similar body weight were put into plastic containers and sent from Japan to our laboratory in Krakow (Poland), where they were used by our team in all experiments from 2015 to 2017 years.

Three kinds of water differing in ion content were used for the larva rehydration process: distilled water, mineral water “Cisowianka”, and tap water, because there were indications that the influence of some physical and chemical conditions of water on some fly larvae^39–41^. Transport across membranes and specific water channels may be dependent on the ion content. For this reason, the content of magnesium and calcium ions was chosen to differ by the order of magnitude (see Table 1).

**Table 1:** Potential of hydrogen (pH) and chemical composition of various types of water used for hydration of larvae *Polypedilum vanderplanki* under laboratory conditions; tap water from “Raba intake” in Kraków; mineral water "Cisowianka".

Parameters	Tap water	Mineral water
pH	7.76	6.80
Oxidation from KMnO_4_ (mg/dm^3^)	0.015	0.027
Chloride (mg/dm^3^)	20.6	< 5
Ammonium ion (mg/dm^3^)	0.05	0.06
Nitrite (mg/dm^3^)	< 0.01	0.013
Nitrates (mg/dm^3^)	4.9	2.4
General hardness (mg/dm^3^)	160	430
Calcium (mg/dm^3^)	40.1	130.3
Magnesium (mg/dm^3^)	6	21.9
General iron (mg/dm^3^)	0.04	< 0.02
Sulfur (mg/dm^3^)	30	< 10
Aluminum (mg/dm^3^)	< 0.008	< 0.008

Standard distilled water came from the laboratory of the Department of Entomology (Jagiellonian University, Krakow). Drinking mineral water was commercially available. Tap water in the sewage system in the laboratory comes from the “Raba intake” in Kraków, Poland. Chemical composition of all kinds of water were analyzed and the results of that analysis are in the Table 1.

### Microtomography

The single *P. vanderplanki* larvae were used for micro-CT scanning analysis to precisely visualize the changes qualitative in the internal body of larvae in the state of anhydrobiosis and the changes during the rehydration process. Anhydrobiotic larvae were analyzed without any modification. Larvae rehydrated in tap water were removed from water after following stages of hydration course: 30 min, 60 min, 80 min, 120 min, and 180 min (active), and subsequently soaked in oil. Immersing in edible sunflower oil was worked out as a method of larva protection from drying out during micro-CT scan. Next, they were placed on pads and covered with a solution of cyclohexanol and formvar in order to prevent drying during measurements. In the presented analysis the scanner Skyscan 1172 X-ray computed microtomograph, SkyScan, Belgium was used. The overall scanning parameters were set as follows: X-ray energy of 40 keV, current 100 µA, rotation step of 0.6°. In the resultant images pixel size was equal 6 µm and was averaged over 10 frames for each image projection. For each rotation angle, the shadow projection was acquired by 11 megapixels CCD flat panel detector. Scanning time was optimized to 32 min per sample, to prevent motion artifacts caused by sample drying. Image reconstruction and analysis were performed using the SkyScan software package (Nrecon v. 1.7.1.0 and CTAnalyser v. 1.16.1.0, Bruker microCT, Kontich, Belgium).

### Scanning electron microscope (SEM)

Using SEM to visualize the changes qualitative in the external body of *P. vanderplanki* larvae in the state of anhydrobiosis and the changes during the rehydration process. Anhydrobiotic larvae, and rehydrated in tap water larvae removed from water after following stages of hydration course: 60 min, 150 min, 180 min (active) were subsequently subjected to the following experimental protocol for observation using SEM. For observation of sleeping chironomid larvae using SEM, the following experimental protocol was applied. The single samples were fixed in 2.5% glutaraldehyde GLU in 0.1 M cacodylic buffer BC for 2 h, rinsed with PBS 2–10 min, and dehydrated in a graded series of alcohols. Finally, they were placed in a transitional liquid (100% acetone) and transferred to a Critical Point Drier (CPD E3000/E3100, Quorum Technologies). Specimens were then coated with gold using a JFC-1100E Ion sputter (JEOL). For coating, the material was placed on a holder with conductive carbon adhesive tabs. Photographs of morphological structures were taken by means of Scanning Electron Microscope (SEM), a JSM-5410, Jeol, Japan, operated at an accelerating voltage of 15 kV.

### Nuclear magnetic resonance (NMR)

Samples of hydrated larvae *P. vanderplanki* used for the NMR experiment were in full active life. ^1^H-NMR spectra were collected on Bruker Avance III 300 spectrometer (Bruker Biospin), operating at the resonance frequency 300 MHz (at *B*_*0*_ = 7 T), with the transmitter power used equal to 400 W. The pulse length was *π/2* = 2.2 μs (corresponding to wide excitation bandwidth of 300 kHz) and repetition time was 3 s. Under this experimental conditions it was possible to examine both anhydrobiotic and live larvae. The data obtained were analyzed using commercially available software Origin 9.0, OriginLab Corporation, USA.

### Morphometry

Anhydrobiotic larvae were placed in Petri dishes, and flooded with various types of water (Table 1). For morphometric measurements we used a stereoscopic microscope NIKON SMZ1500, Nikon, Japan, with a digital camera. NIS-elements BR software was used to archive images and body measurements. The larvae from three containers with serial numbers: 2, 8, 32 were used.

At the beginning of the procedure, dried larvae were photographed. Subsequent photos were made and recorded after every 2 min until the larva was fully hydrated, which was indicated by its vigorous activity as: the movement of all parts (head, thorax, abdomen) of the body and of its appendages. Dorsal view of larvae were photographed. Each photo was taken at an A1x magnification and at the resolution of 2560 × 1920 pixels. The area was determined on the basis of the larva's body color different from water. Changes in the surface of the body observed larvae were measured by comparing size measurements of successive steps during hydration with respect to the first measurement. For each type of water (distilled, tap, mineral) this procedure was repeated three times for every single simple.

Data records of surface measurements were archived in the database made in Ms Office Excel and after that were analyzed using the OriginPro 2015 program.

## Results

**Figure 1:**
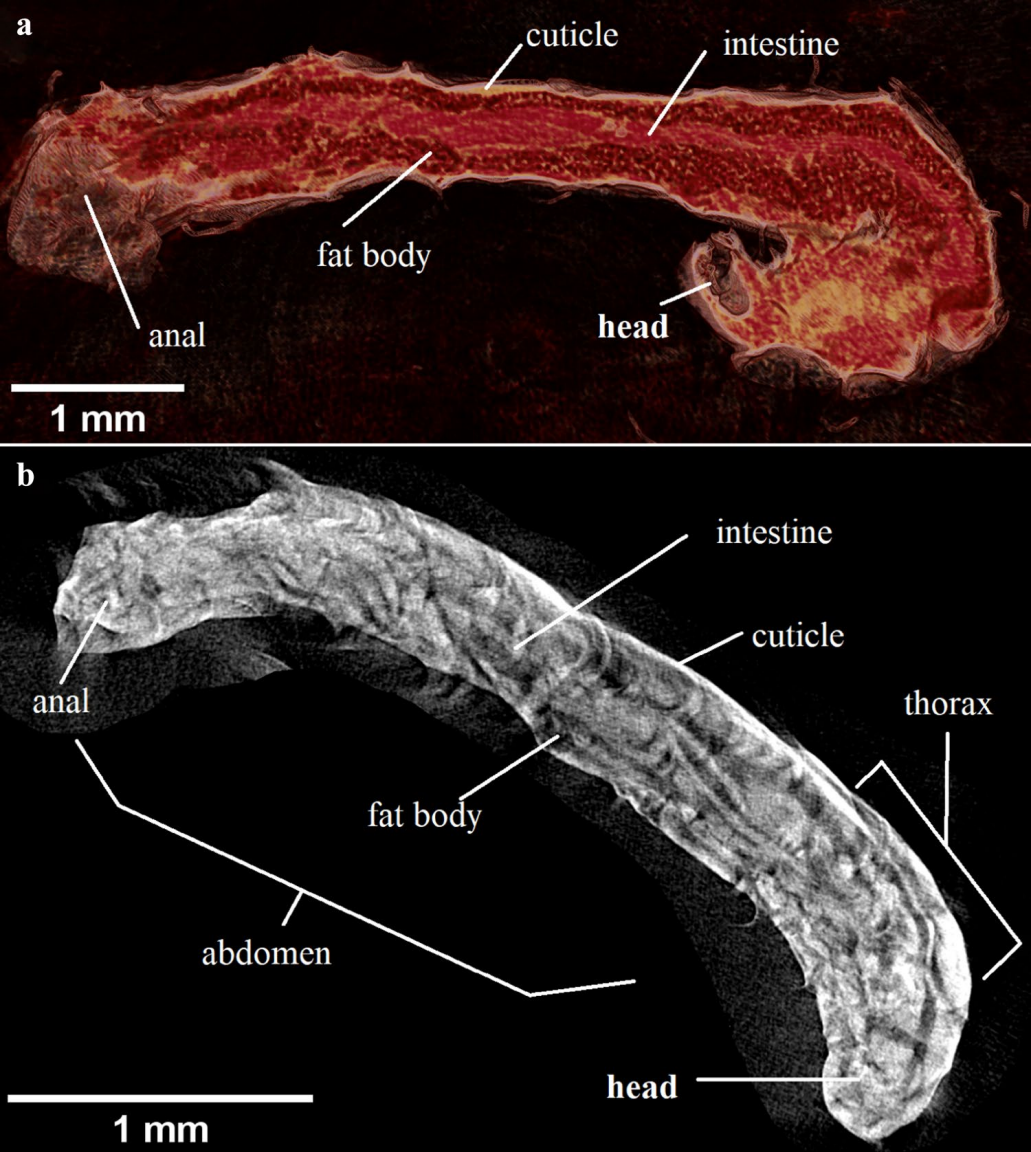
The larvae *Polypedilum vanderplanki* (longitudinal view, micro CT), visible: head (right side of larvae at the bottom), cuticle—lighter outer line, fat body—smaller granulation under the cuticle, intestine filled with content—lighter color; (**a**) after 80 min of hydration, and (**b**) after 120 min of hydration.

Microtomographic measurements of *Polypedilum vanderplanki* showed morphological patterns of anhydrobiotic larvae and captured their morphological changes in course of the rehydration process. Photos of these larvae were taken along the body (Fig. 1).

Figure 1a shows the external (head with mouth, thorax, abdomen with anal and cuticle) and the internal (fat body and intestine) morphological body parts of the larva after 80 min of hydration.

Figure 1b presents a larva after 120 min of hydration. Lower levels of water absorption into the larva body indicate darker places, whereas higher water absorption levels—the lighter places (Fig. 1b). The highest variability in the absorption level of water is observed in the central regions of the larva body, where the most physiologically active organs, such as: the central part of the intestine together with Malpighi tubes, reproductive organs and the dorsal vessel (insect heart) occur.

**Figure 2:**
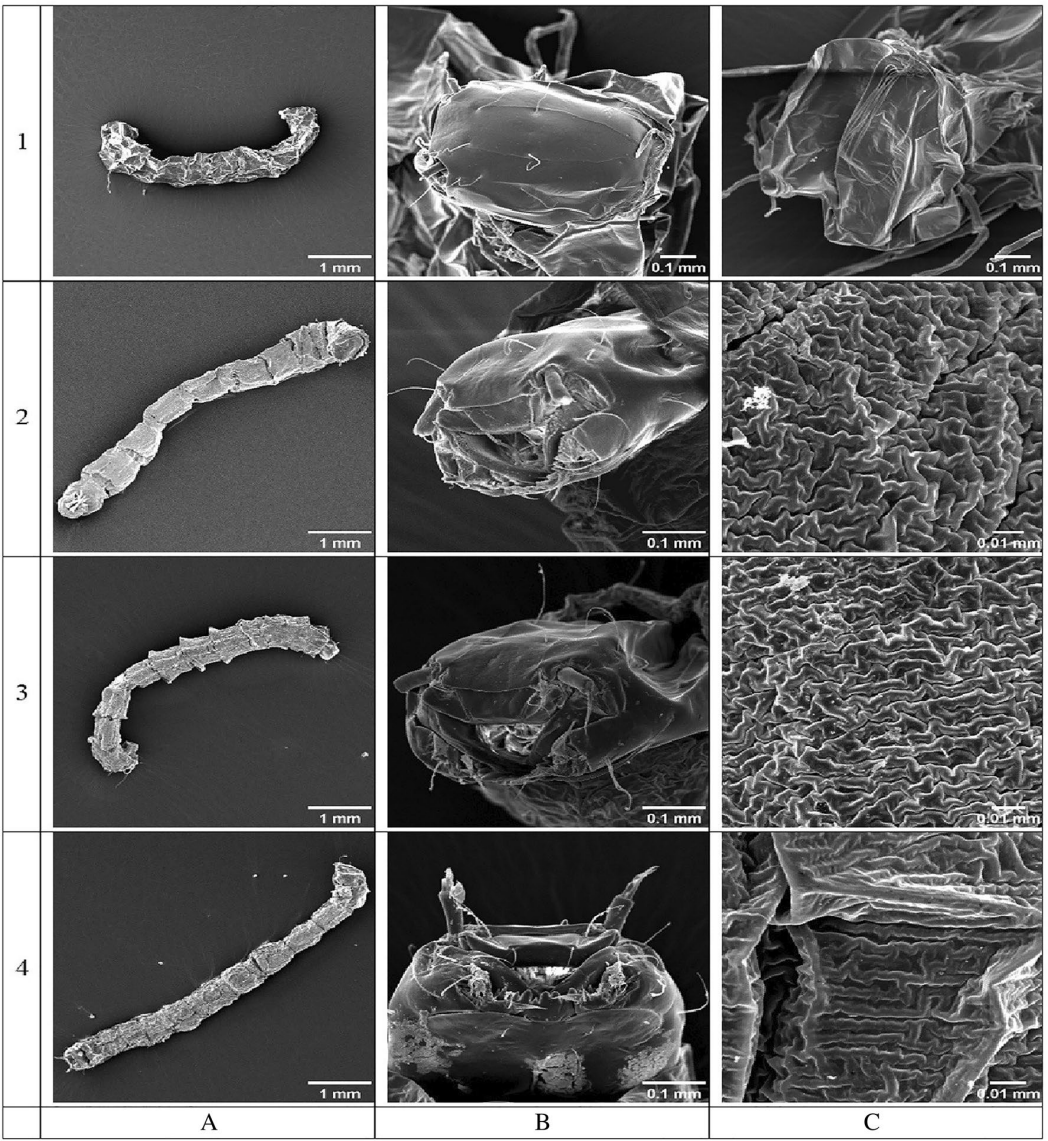
Body structure of *Polypedilum vanderplanki* larvae at different stages of hydration in tap water: (**A**)—habitus (SEM, ×35), (**B**)—head (SEM 1—×200, SEM 2–4—×350), (**C**)—surface of abdomen (SEM 1—×200, SEM 2–4—×2000); 1—anhydrobiotic larva, 2—larva after 60 min of hydration, 3—larva after 150 min of hydration, 4—larva after 180 min of hydration (full hydrated, active larva).

The SEM micrograms demonstrate morphology of the anhydrobiotic *P. vanderplanki* larvae and changes in body structure of a larva during the hydration process performed in tap water (see Fig. 2).

Body of dehydrated larva is rigid, slightly curved, elongated, and wrinkled, without apparent segmentation (Fig. 2, 1), which is usually characteristic for the free-living larvae of many different fly families, particularly for Chironomidae.

**Figure 3:**
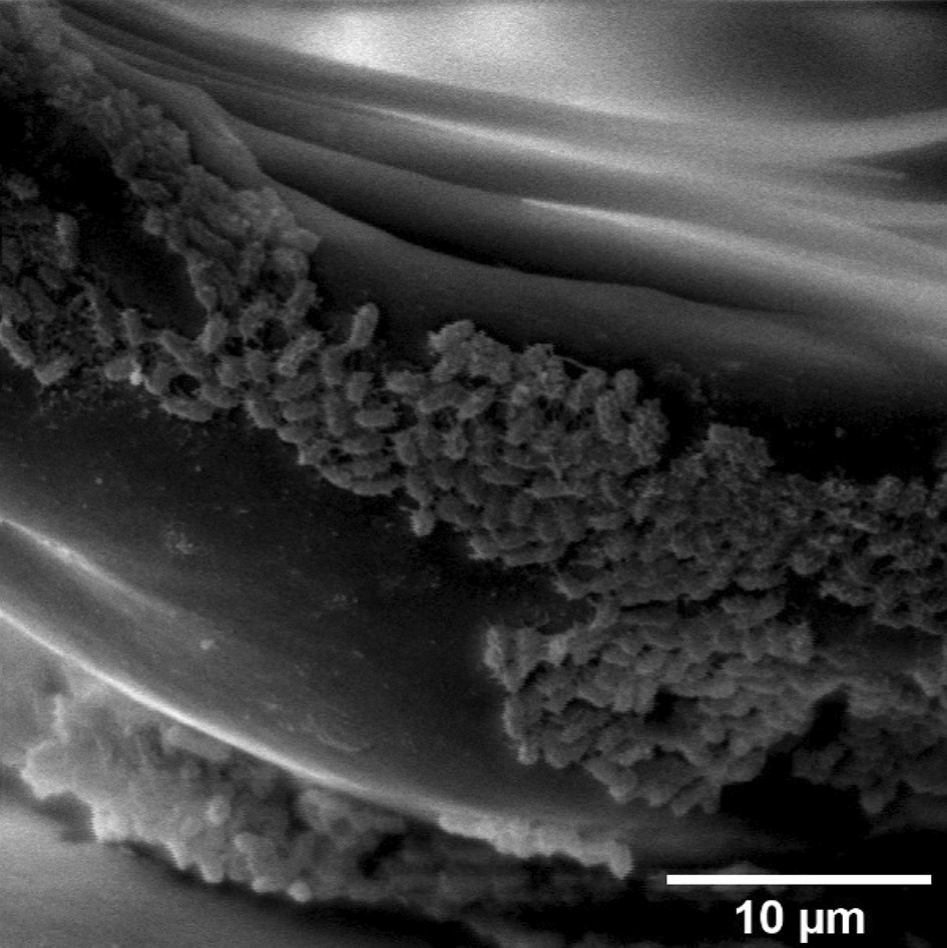
Bacteria or fungal spores visible on the *Polypedilum vanderplanki* larva head of after 150 min of hydration course (SEM, magn. ×5000).

With the hydration level increase, head and body segmentation becomes more and more visible, the cuticle is more elastic, and the shape of the larva is more upright (Fig. 2, 2–4). The head structure of larvae does not change much during hydration (Fig. 2B, 1–4) because its cuticle includes much of hydrophobic chitin, and is relatively thick, rigid and hard. Probably the head does not absorb at all or absorb only small amounts of water through its cuticle. Water enters the body of the larva mainly through the mouth and anus, and a thinner layer of cuticle surrounding its thorax and abdomen (Figs. 1, 2A 1–4,C 1–4), which is confirmed by e.g.: Beament et al., Buck, Verhoef and Witteveen, Block and Harrisson, Danks^4,42–45^. On the head of hydrated larva we also detected some microorganisms, reminding cultures of bacteria, or fungal spores (Fig. 3).

**Figure 4:**
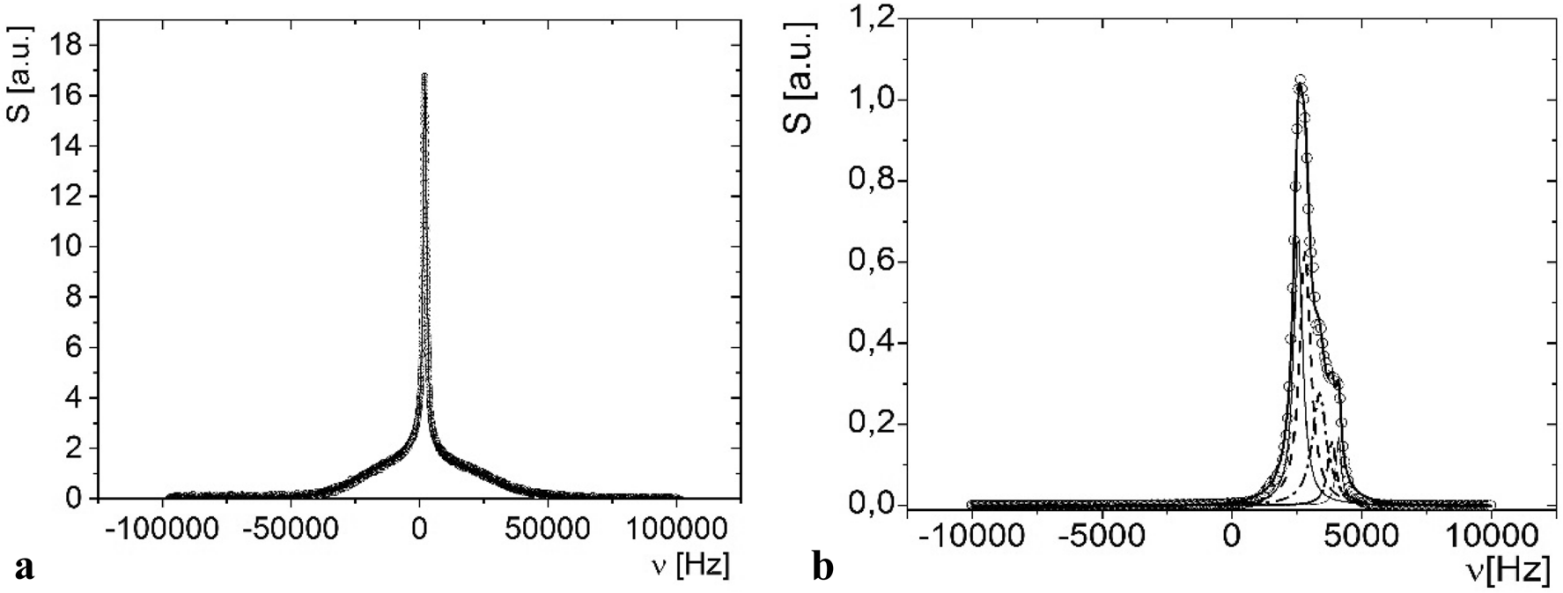
^1^H-NMR spectra for larvae of *Polypedilum vanderplanki*: in (**a**) anhydrobiotic form (a sample consisted of ca. 140 individuals), (**b**) active form (a sample consisted of 5 specimens); both recorded at 300 MHz, the pulse length *π/2* = 2.2 μs; *OX* frequence, *OY* amplitude.

The ^1^H-NMR spectra for anhydrobiotic (a) and in active life state (b) larvae of *P. vanderplanki* were recorded at 300 MHz (Fig. 4).

Anhydrobiotic larva may be treated as a microheterogeneous system consisting of significantly immobilized proton component, for which the local magnetic fields do not average, and the mobile proton component with local magnetic fields averaged (Fig. 4a). Thus, the detected spectrum is a superposition of the broad line coming from the solid matrix of dehydrated tissue, which is successfully fitted by a Gaussian function, and one averaged narrow component, which comes from water bound and/or from lipids of anhydrobiotic larva, fitted well by a single Lorentzian function, that Eq. (1) is:

1 $$S\left(\nu \right)=\frac{{S}_{S}}{\Delta {\nu }_{G}\cdot \sqrt{\mathit{ln}\,4\cdot \pi /2}}\mathit{exp}\left[-2\mathit{ln}\,4\cdot {\left(\frac{(\nu -{\nu }_{G})}{\Delta {\nu }_{G}}\right)}^{2}\right]+\frac{2{S}_{L}}{\pi }\left[\frac{\Delta {\nu }_{L}}{4\cdot {\left(\nu -{\nu }_{L}\right)}^{2}+\Delta {\nu }_{L}^{2}}\right],$$

where $\Delta {\nu }_{G}$, and $\Delta {\nu }_{L}$ are the half-widths of the NMR line components; *v*_*G*_, and *v*_*L*_ are the peak positions; and finally S_*S*_ and *S*_*L*_ are the amplitudes of the Gaussian and Lorentzian peak, respectively. The values of parameters of fitted line components are set up in the Table 2.

**Table 2:** The values of parameters of analyzed ^1^H-NMR spectra recorded for anhydrobiotic and natively hydrated (active, live) larvae of *Polypedilum vanderplanki*; areas under lines are normalized to the total area under each spectrum.

	Area under line, *S*_*G/Li*_	Line half-widths, $\Delta {\nu }_{G/Li}$	Peak position, ${\nu }_{G/Li}$
**Anhydrobiotic**			
Solid component	*S*_G_ = (70.1 ± 1.4) %	Δ*v*_G_ = (45,654 ± 93) Hz	*v*_G_ = (1880 ± 32) Hz
Liquid component	*S*_L1_ = (29.885 ± 0.038) %	Δ*v*_L1_ = (1582.0 ± 2.6) Hz	*v*_L1_ = (2042.47 ± 0.81) Hz
**Active**			
1st liquid component	*S*_L1_ = (31.3 ± 5.3) %	Δ*v*_L1_ = (422 ± 24) Hz	*v*_L1_ = (2548.5 ± 9.9) Hz
2nd liquid component	*S*_L2_ = (36.4 ± 8.2) %	Δ*v*_L2_ = (521 ± 59) Hz	*v*_L2_ = (2816 ± 16) Hz
3rd liquid component	*S*_L3_ = (20.7 ± 5.3) %	Δ*v*_L3_ = (670 ± 110) Hz	*v*_L3_ = (3371 ± 18) Hz
4th liquid component	*S*_L4_ = (6.9 ± 3.3) %	Δ*v*_L4_ = (390 ± 120) Hz	*v*_L4_ = (3852 ± 21) Hz
5th liquid component	*S*_L5_ = (4.7 ± 1.4) %	Δ*v*_L5_ = (245 ± 44) Hz	*v*_L5_ = (4112 ± 12) Hz

The half-width of the solid line component is equal to $\Delta {\nu }_{G}$ = 46 kHz, and a position of a broad peak is ${\nu }_{G}$ = *ca*. 2 kHz.

The ^1^H-NMR spectrum, recorded for active larvae, indicates drastic changes, compared to that for anhydrobiotic ones. The solid signal component is no longer fitted, and the spectrum is well described by the superposition of five line components coming from different fractions of mobile protons (see Fig. 4b). The spectrum is fitted well using a superposition of five Lorentzian functions (2), according to:

2 $$S\left(\nu \right)={\sum }_{i=1}^{5} \frac{2{S}_{{L}_{i}}}{\pi }\left[\frac{\Delta {\nu }_{{L}_{i}}}{4\cdot {\left(\nu -{\nu }_{{L}_{i}}\right)}^{2}+\Delta {\nu }_{{L}_{i}}^{2}}\right],$$

where $\Delta {\nu }_{Li}$ are the half-widths of the NMR i-th line component; ${\nu }_{Li}$ is its peak position; and ${S}_{Li}$ is the amplitude of i-th Lorentzian peak.

**Figure 5:**
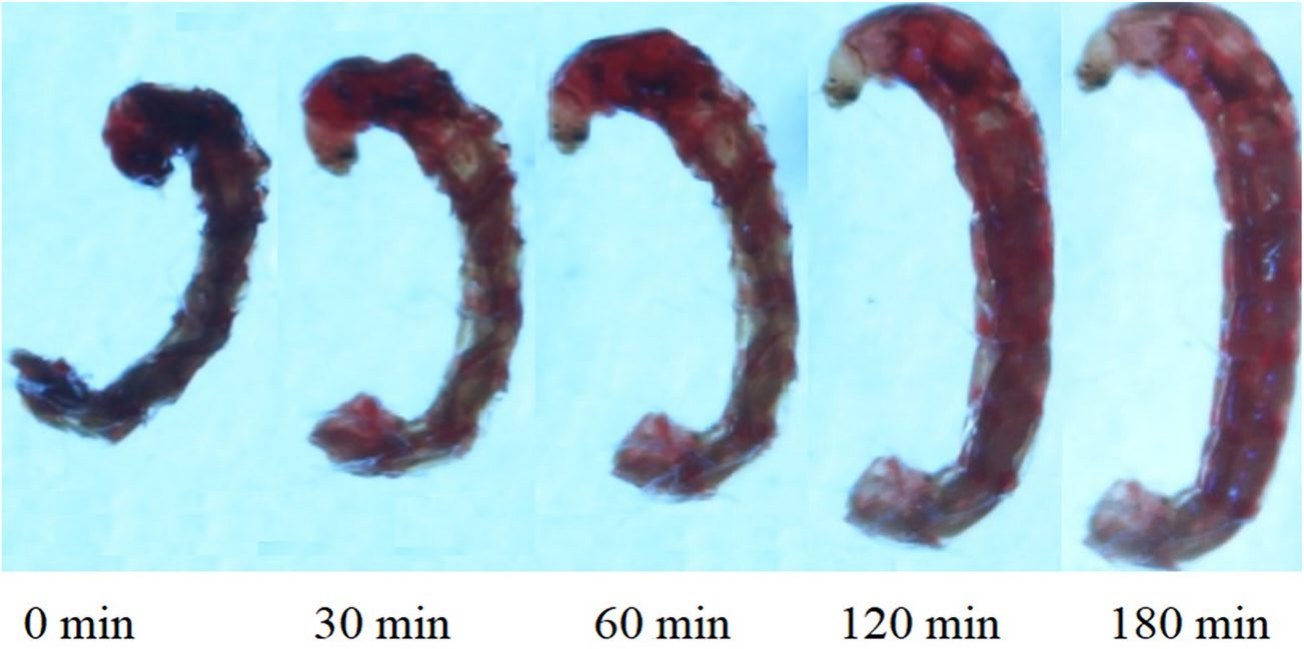
Sagittal views of *Polypedilum vanderplanki* larvae in the stereoscopic microscope NIKON SMZ1500 after putting in the water (0 min), and after 30 min, 60 min, 120 min, 180 min of rehydration.

For description changes of the surface of each analyzed live (Fig. 5) and dead larvae during hydration, we used the allometric function (3):

3 $$A\left(t\right)={A}_{0}+{b(t)}^{c},$$

where *t* is the hydration time, *b* and *c* are the fit parameters, *A*_*0*_ is 100 because the surface of the dehydrated larva was taken as 100%. The *c*-factor was a measure of the method of hydration, indicates a deviation from the linearity of the hydration process. An allometric function has been used to describe disproportionate relations between selected quantities characterizing e.g. the structure of an organism^46–48^.

During the rehydration process of the larva its surface (*A*) has been increased according to the following form of power law: *A* = *const·t*^*2/3*^.

**Figure 6:**
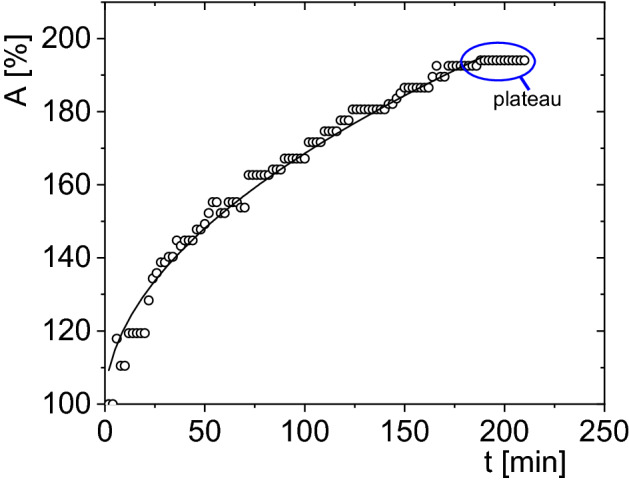
The increase of the surface area, A, as a function of time, t, for *Polypedilum vanderplanki* larva`s body; hydrated in distilled water after the first day of opening the container No. 8 (matching parameters: b = 6.48 ± 0.31, c = 0.51 ± 0.10), open circles—data points, solid line—fitted allometric function (Eq. 3). The experimental errors are within the size of the symbol.

Knowing that also *A* = *const* · *V*^2/3^, where *V* is volume of larva, and this constant is characteristic for a particular shape of a measured object, it may be concluded that the volume of larva *P. vanderplanki* during rehydration increases proportionally to the time course (Fig. 6). The results were expressed as a percentage in order to compare with each other all increases in the surface of tested larvae. Figure 5 shows the hydration results for a single larva. A plateau in size increase was observed after the time value (170 min) when the larva became fully rehydrated and revealed vital activity. This time was defined as medium activation time (MAT). An average of MAT for all analyzed larvae when plateau occurs is 157 min.

**Figure 7:**
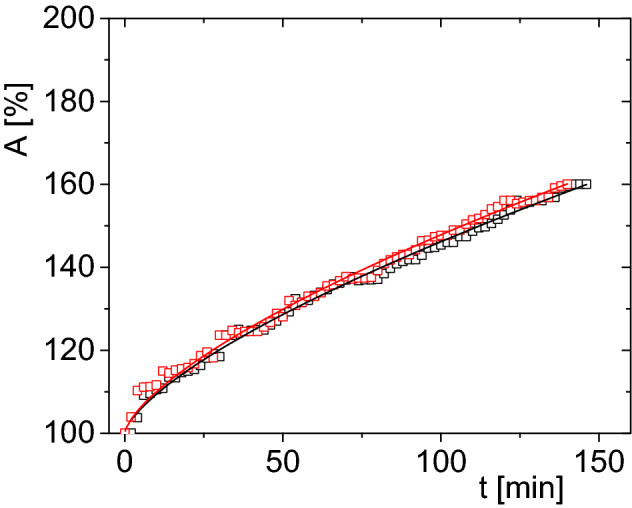
The increase of the surface area, A, as a function of time, t, for *Polypedilum vanderplanki* larva`s body; hydrated in tap water after the first day of opening containers: red squares—larvae from container No. 3. Solid line—fit of Eq. (3) (matching parameters: b = 2.23 ± 0.10, c = 0.66 ± 0.01), black squares—larvae from container No. 8 (matching parameters: b = 2.21 ± 0.09, c = 0.66 ± 0.01). The experimental errors are within the size of symbol.

**Figure 8:**
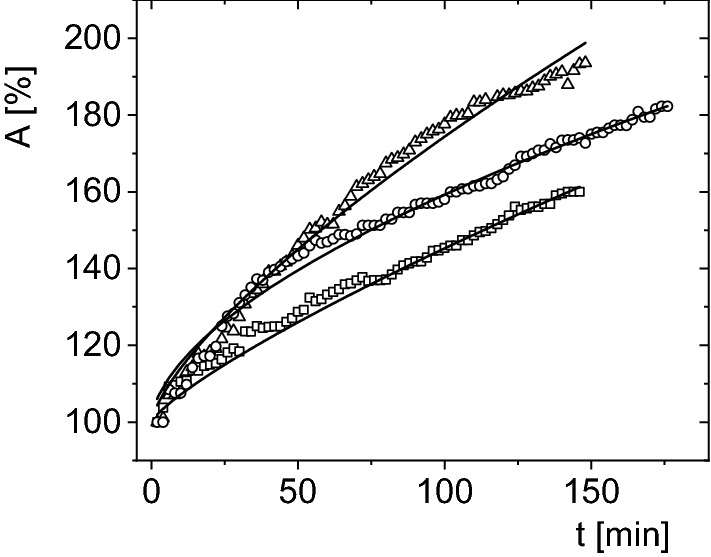
Stacked plot of increase in the surface area, *A*, expressed as a function of time, *t*, for *Polypedilum vanderplanki* larvae hydrated in various types of water: open circles—distilled water, open squares—tap water, open triangles—mineral water; larvae taken for analysis from the same container (No. 8) after one day of the first opening.

Based on the morphometric data, models of surface increments were constructed for each type of water used for hydration of larvae. The results indicate that the hydration process in the same type of water is described by a similar functions regardless from which sample the analyzed larvae came from (Fig. 7). However, models of surface increments for larvae hydrate in various types of water are different (Fig. 8).

Table 3 shows the parameters for *P. vanderplanki* hydration courses fitted using Eq. (3). To correct the obtained values, the function from Eq. (3) was fitted to each hydration course separately, and subsequently the mean value was calculated (Table 3). These fits are similar to each other, although they slightly differ. The value (0.66 ± 0.02) of coefficient *c*, showing the observation method on the microscope screen, means that the increase in volume of hydrating larvae over time is linear. No threshold changes were detected. It means that spiracles, being natural openings in the body of the larva, have no significant effect on the hydration process. Probably only mouth and anal opening play the crucial role in this process.

**Table 3:** Medium activation times, MAT, of *Polypedilum vanderplanki* larvae after hydration courses in various types of water; b and c—adjustment coefficients of allometric functions Eq. (3); total—average of all measurements.

Water type	Individual measurements	Matching to models			
	MAT (min)	b	c	b	c
Distilled	174.50 ± 44.43	4.06 ± 2.40	0.57 ± 0.07	4.10 ± 0.23	0.58 ± 0.02
Tap water	145.20 ± 20.03	2.74 ± 0.84	0.66 ± 0.14	2.21 ± 0.09	0.66 ± 0.01
Mineral	150.00 ± 17.20	2.79 ± 2.05	0.75 ± 0.12	2.65 ± 0.18	0.75 ± 0.02
Average	156.57 ± 27.22	3.53 ± 1.76	0.66 ± 0.11	2.99 ± 0.17	0.66 ± 0.02

In the initial phase of the hydration process, about 20 min after beginning of the hydration course, no differences between the larvae hydrating in mineral and distilled water were detected. In contrast, hydration of larvae in tap water differs from the other two experiments. However, after *ca*. 30 min of hydration course the differences between the larvae hydrated from mineral and from distilled water appear (Fig. 8). Compared to those (Table 1), at the hydration course performed from tap water the volume of larvae increases slowly, also the total level of hydration is lower (*A* = 163%), and MAT is shorter (145 min). In addition, for this case the exponent of the allometric function, *c* = 0.66, and the water volume increase in the larva body is almost linear. This indicates that at tape water hydration course anhydrobiotic larvae need least time and the least amount of water absorption for rehydration to fully active form. It seems that such a hydration course reflects the best the rehydration process occurring in natural environmental conditions (water scarcity) where larvae of *Polypedilum vanderplanki* do exist.

For larvae hydrated in distilled water, MAT is equal *ca*. 175 min, what is the highest value as compared with hydration processes from other water types, and the exponent *c* = 0.58 (Fig. 8). However, the final hydration level (A = 182%) is higher than that in tap water but lower than in mineral water. The hydrated from distilled water larvae need more time to obtain their full life activity and absorb it slowly because this type of water contains lesser amounts of ions relative to other types of water (Table 1).

In turn, the hydration course of larvae from mineral water indicates the highest value of the allometric exponent (*c* = 0.75) and also the level of hydration is highest (*a* – 193%) with a relatively short MAT (148 min), as compared to hydration kinetics from other of water types (Fig. 8). The obtained difference in the value of the coefficient c may also result from the inaccuracy of the measurement method. At hydration courses from mineral water larvae hydrate easily, absorb water faster returning in a short time to complete life activity. Mineral water contains the rich set of ions, and particularly the highest contribution of Calcium and Magnesium ions (Table 1) what makes it easier and faster the opening of water-permeable channels during the larva hydration course than in mineral-poorer waters condition such as distilled water.

**Figure 9:**
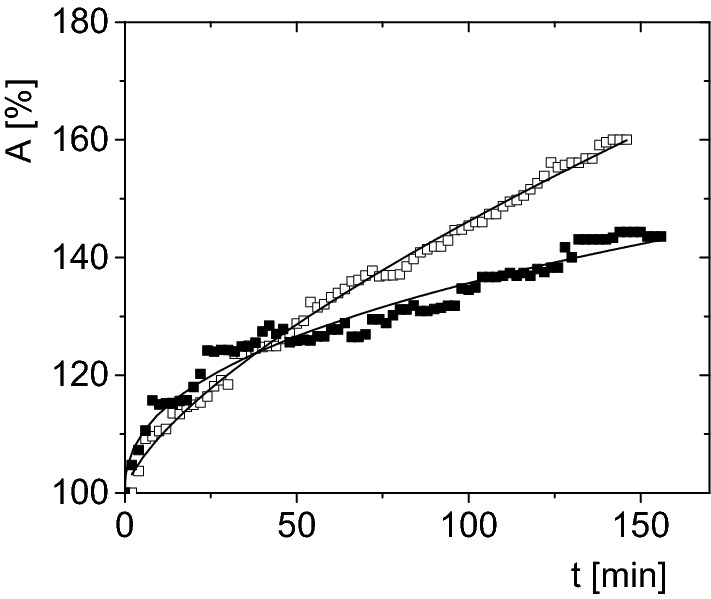
Stacked plot of increase in the surface area, *A*, expressed as a function of time, *t*, for *Polypedilum vanderplanki* larva; hydrated in tap water after the first day after opening of the container: open squares—alive larvae from the container No. 8, full squares—dead larvae container No. 32; continuous line—fit to Eq. (3).

Hydration measurements of dead larvae were also performed (Fig. 9) to check whether the biological functions of live larvae affect the hydration process under the same conditions. Difference in the slope of the fit to the measuring points for dead larvae suggests a different hydration mechanism (Fig. 9). The volume increases at hydration courses for alive and dead larvae are similar up to *ca*. 50 min of hydration, but for longer times of rehydration they differ. In the case of live larvae there is a faster increase in body surface area (*A*) at the same time than that at hydration course for a dead larva (Fig. 9). This indicates that in the initial stage of the hydration process, water penetrates into the larva's body mainly through the mouth and anus either for alive or for dead larva. Then, in living larvae, tissues are probably activated and water-permeable channels are launched, and water enters the body faster and in greater volume.

## Discussion

The obtained microtomograms of *Polypedilum vanderplanki* larvae recorded during hydration courses revealed the changes in external and in internal morphology depending on the duration time of this process. As the rehydration proceeds, the larvae become better and more clearly visible in their individual external body structures. After complete rehydration, the larva becomes vital and all parts of its body are visible, as described in the larvae before the process of anhydrobiosis.

To our best knowledge, the SEM studies presented here are the first which present changes in the morphology and the body structure of the anhydrobiotic larvae *P. vanderplanki* during the rehydration process in liquid water (Fig. 2). SEM micrograms indicate that the head structure of larva does not change much during the rehydration course which is in contrast to the thorax and abdomen, whose structure of the body surface clearly changes during this process. This study helped us not only to know how the body of the larva behaves during rehydration but also to discover some microorganisms on this larva head (Fig. 3). We are not sure what it is. It was difficult to determine at present study whether they are bacteria or fungal spores, and whether they accompany the larvae's life, or it is an artifact and these microorganisms could have come from the outside during the dehydration, rehydration or transport process. However, given the size of the objects, their appearance and location, it seems that they are most likely microorganisms. Current data indicate that many taxa of microorganisms (mainly bacteria) associated with larvae stage of *P. vanderplanki*, also anhydrobiotic^49^. Microorganisms inhabiting larvae of the anhydrobiotic midge can also sustain desiccation^50^. It is also known that the larvae of *P. vanderplanki* eat also protists, algae and cyanobacteria^51^. In the digestive tracts of some Chironomidae larvae microorganisms community has been found^52^. The ^1^H-NMR immobilized proton signal component coming from the tissues of anhydrobiotic larva does not differ much from the one detected for other microheterogenous solids, and particularly, for other deeply dehydrated biological systems, eg. DNA-surfactant complexes^53^, photosynthetic membranes^54^, or for anhydrobiotic living organisms, e.g. Antarctic lichenized fungi^55–57^. The observed mobile proton signal may be assigned to residual water^54^ bound in dry larva, and/or to lipids mainly localized in larva fat bodies.

The mobile proton signal detected for anhydrobiotic *P. vanderplanki* larva was relatively high, which suggests that despite of the stopping of life activity its organism deposits noticeable water reservoirs inside its body. The access of lipid fraction probably is negligible, as it was not observed at FID and CPMG experiments of ^1^H-NMR relaxometry (e.g.^58^) for dehydrated larva.

Most likely the detected ^1^H-NMR spectra for active, but rehydrated from anhydrobiosis, *P. vanderplanki* larva mainly present the signal from tightly bound water fraction, and from loosely bound water fraction, as well as signal from lipids. The linewidths for *L*_3_, and for *L*_2_ components suggest that at least *L*_3_ line component comes from tightly bound water fraction. Other line components come from intra- and extracellular loosely and/or free water fractions, and from water deposited in guts of larva. The activation time is relatively short for significant metabolic synthesis of large amounts of lipids in the larval body, thus, the observed lines come from several water fractions.

The solid matrix of the *P. vanderplanki* tissue was probably partially hydrated, or at least remained unchanged in magnitude, whereas the mobile proton signal drastically increased at rehydration process. It resulted in a completely different form of ^1^H-NMR spectrum detected for active rehydrated from anhydrobiosis larva, in which the remaining part of immobilized proton signal is no longer fitted.

The cuticle forming outer integument of Insecta body is varied in internal structure and provides a very solid, efficient and lightweight skeleton and prevents the organs from environmental stresses, among them desiccation shock and decreased temperature (e.g.^54,58–61^). Insect cuticle plays also important role in controlling water level in the insect body, especially for water insects in liquid permeability, although water into it inside the body can also be taken up through the rectum against large concentration gradients or by the mouth, anal opening, as well as with food (e.g.^62^). In the cuticle and in the rectum instances, there is an active transport of water without the transport of other materials, which indicates that the cuticle is perfectly semipermeable. The absorption of liquid water into the cuticle is accompanied by temporary reorganization of lipid molecules, and the cuticular protein being under an active control by the epidermis can also modify the hydration of the cuticle^42^. In the larvae of the sleeping chironomid, *P. vanderplanki* is a channel protein called Aquaporin that allows water to permeate across cell membranes, however, the mechanism by which water flows out of the larval body during dehydration is still unclear^14^. The mechanism of water entering the body of the larva during its hydration from anhydrobiosis is also unknown. No slowening, or any threshold changes in this process were detected. Such a character of the dependency suggests that during the imbibition process its effectiveness remains mostly unchanged, as it is in case of the hydration occurring by a diffusion process and through the natural body holes, e.g. the mouth and anal opening. Moreover, the natural body holes do not stretch significantly during hydration. The initial hydration level is common to all hydration courses investigated, so it can be assumed that it is not dependent on the kind of water used in the experiment. It is possible that the transport of water occurs spontaneously or the larva takes water through the body's holes. However, water transport through channels, which is associated with ions, is additionally activated. It seems that the chlorine content in tap water is responsible for the change after 50 min of hydration, whose content compared to mineral and distilled water is much smaller. In the case of mineral water, ions are responsible for increased volume of water penetrated into the body of the larva, while in tap water this permeability is lesser. The distilled water experiment shows the hydration process in case of an insufficient level of ions in the external environment of the larvae. The individual differences between the larvae and the standard deviations of the calculations indicate that during the hydration of the larvae in different types of water with different chemical composition and ions contents, the hydration courses do not differ significantly. In each type of these water kinds, larvae can be restored to full life activity, which means that they are resistant to fluctuations in the chemical composition of water. It is likely that larva fit to anhydrobiosis induce mechanisms that modify the permeability of the cuticle and epidermis during hydration which dead larva cannot. This reflects the differences in the method of hydration between live and dead larvae under the same water conditions. It can be assumed that channels and diffusion play a key role in the permeability of water by the body integument of the larvae. Water probably gets inside the dead larvae mainly by diffusion, because without triggering any vital processes, they are not able to activate another way of transporting water through the cuticle. Nor can they suck water in through their mouths as live larvae do.

## Conclusions

There are no threshold changes during the hydration of *P. vanderplanki* larvae (Figs. 6, 7, 8, Table 3). The value of the coefficient c indicates a linear nature of the increase in the volume of live hydrated larvae from the liquid phase over time. No change/gradual changes/sudden changes during rehydration is a sign that it is evenly process.

Microtomographic analyzes using a stereoscopic optical microscope (SOM) and a scanning electron microscope (SEM) were consistent. The body of *P. vanderplanki* larva is differentiated into a clearly visible head, 3 segmented thorax and multisegmented abdomen. During hydration, the volume of the larva's head hardly changes, while the remaining parts of the body increase in volume, the larva body stretches and lengthens, and the outer surface of the cuticle becomes less and less wrinkled.

The larvae are resistant to fluctuations in the chemical composition of the water. The initial hydration phase does not depend on the chemical composition of water, but the amount of ions present in the medium affects the further process and the rate of larva's return to active life. Ion channels and diffusion play a major role in the permeability of water through the larva's body integument.
